# Gout

**Published:** 1892-09

**Authors:** 


### GOUT.

Acquired gout is usually one of the consequences of errors and excesses of diet. Those who eat too much meat and drink too much wine are, as is well known, very frequently the subjects of the disease. But it is by no means so well known that beer is a prolific cause of gout. The fact is so, however. Dr. Frederick Roberts in "Quain's Dictionary of Medicine," tells us that brewers' draymen are particularly subject to gout. Malt liquors, Dr. Roberts considers, stand next to wines as originators of gout. Good whisky and brandy, on the other hand, are said to be much less mischievous in this connection. Brewers' draymen, though comparatively poor in pocket, do not generally suffer from poor man's gout. On the contrary, their gross and ponderous bodies are gorged with the products of their own excesses. Some of them, it is said, drink as much as two to four gallons of beer a day. Sir Alfred Garrod, a competent authority, states that lead taken into the system is a potent cause of gout. No less than 30 per cent. of Dr. Garrod's hospital patients owed their gouty seizures to working among lead. Many of these were probably physiologically poor, poor in blood and tissue; and they would, no doubt, suffer from what is popularly called poor man's gout. Butchers and barmen, coalheavers and painters, and others who have to do with lead, are all liable to the disease.

